# The impact of instructing management skill to managers on the obstetrician’s efficiency

**Published:** 2015

**Authors:** B Maleki, F Rahimikian, T Salehi, A Mehran

**Affiliations:** * Department of Midwifery, School of Nursing and Midwifery, Tehran University of Medical Sciences, Tehran, Iran; ** Faculty of Nursing and Midwifery Care Research Center, School of Nursing and Midwifery, Tehran University of Medical Sciences, Tehran, Iran; *** Department of Internal Surgery, School of Nursing and Midwifery, Tehran University of Medical Sciences, Tehran, Iran; **** Faculty of Biostatistics Department, School of Nursing and Midwifery, Tehran University of Medical Sciences, Tehran, Iran

**Keywords:** instructing, management skill, obstetrician’s efficiency, managers

## Abstract

The importance of efficiency and improvement of health service for resolving people’s health requirement and meeting their expectation is increasing. In addition, it considers as a priority for making decision and manager’s activity in health officials. Manager’s control on the management principle and the proper use of their management skill and creating a sense of trust and commitment are the tools that were providing a good condition for working and catching the organization’s goals. In this quasi-experimental study, before beginning the research, the non-teaching hospitals that are affiliated to the Kurdistan’s medical science university were randomly divided into 2 groups. Three hospitals from 3 cities considered as a control group, and three hospitals from 3 cities considered as an intervention group.80 person of hospital’s obstetrician staff classified in these 2 group by quota method and the hospital’s nurses and obstetrician’s manager of case-control involved by census method. The research’s tool was Hersi and Gold Smith’s standard efficiency questionnaire, which was filled out at the beginning of the study by the obstetricians of both groups and then it gave to the hospital’s nurses and obstetrician’s managers of the case group’s instructing management skills for 16 hours. The efficiency’s questionnaire was filled out, compared, and evaluated again by the obstetricians of both groups, 12 weeks after intervention. The data analyzed by the independent T-test, variance analysis, paired T-test, and SPSS 22. The findings showed that the average of the obstetrician’s efficiency mainly developed in the intervention team after the instruction of management skills to the managers (P < 0.001). Conclusion: The instruction of the management skill to the nurses and obstetrician’s managers caused the efficiency’s promotion. Therefore, the instruction of the management skills has suggested as a method of increasing efficiency in hospitals.

## Introduction

Efficiency is an overall concept, and the increase of it consider as an essential fact for the promotion of life’s level, more welfare, human’s comfort which is the importance aim of all the countries in the world and always it is an important fact for governments [**1**]. In today’s competitive world, efficiency as a philosophy and a point of view, which was based on the improvement of strategy are one of the primary goals of each organization and like a chain, it could contain the society’s activity in all parts [**2**]. In services, the human gives the services. Therefore, if this man is an incentive, formidable and efficient he could use all his sources appropriately and bring an overall efficiency and finally bring the productivity for the organization. If not, stagnation and backwardness come from a passive manpower [**3**]. The health unit has a vital and stable responsibility toward people during their life, from the healthy infant’s secure birth to caring an elderly with respect [**4**]. Unlike the industrial and commercial organization, the health care team rarely evaluate the efficacy’s increasing methods in their staffs and the researchers which have done by the organization, and mechanical part of the country are not proper for hospitals [**5**]. According to the importance of the manpower in an organization and their role in improvement and meeting the team’s goal, paying attention to the staff’s efficiency is one of the principal concern of the managers. The director’s mission and their primary goal are the optimization use of manpower. Because it is not only a source organization but also it is a factor, which causes the use of other useful factors on efficiency. Running the efficiency improvement program would start at the management level because this is in the manager’s responsibility field and the success and failures of the energy program depend on the attitude, strategy, policy and the director’s action which is more important than the other factors. Managers could affect the personal’s attitude, emotion, thoughts, and more effectiveness in his work condition. He would use the efficiency method. Nevertheless, most of the managers due to the lack of knowledge and not passing the instructing period of management and leadership are acting on their previous experiences in their workplace. In addition, its results could be the low-efficiency expectation of the organization [**6**]. So many describe the active factor of efficiency in a model which constitute of 4-factor technology, material, manpower, and management. Also, Rostas reminds the management proficiency as a primary element in the efficiency. Also Hersi and Goldsmith emphasis on the management’s performance as the central origin of ability by presenting a model [**5**,**6**]. The above research selected seven variables from others, which were related to the efficient management performance and these factors were Ability, Clarity, Help, Incentive, Evaluation, Validity, and the environment. Based on these facts the man’s performance could be evaluated [**7**]. Osoan said that more than half of the nurse managers believed that they aren’t ready enough for management role [**8**]. The manager’s control on their management skills and creating a sense of trust and commitment and the nurses participating and cooperating are the tools, which provide a proper condition for work and catching the organization’s goal. In fact, the increase of the efficiency is a sign of organizing an excellent management and criteria for team’s improvement due to the proper management [**9**]. Dehghannayeri (2006) in his study showed that the nurses believed that the efficiency of them and the nursing team is because of the manager’s knowledge and awareness of management. For this reason, the instruction period of the Directorate holding for all the nurses’ manager and matron is necessary [**10**]. Managers in organizations tried to be successful by coordination and effectiveness [**8**,**9**].

Katz divided the management skills into three groups: 1) technical expertise like being familiar with process and technology application. 2) Perceptual skills like thinking, problem solving, innovation, etc. 3) man’s skills like leadership and human’s relationship, recognition, incentive, etc. the relative importance of these skills are different in the organization’s hierarchy. In each level of the management, the effectiveness and success of the managers depended on the skills, which related to that level [**11**]. Peter believed that the personals efficiency is the first director’s competence test [**12**]. Obstetricians as specialist manpower in health center have an important role in women’s caring, curing and spiritual and physical support. And also they are responsible for maintenance and promotion of infants and mother’s health, and also they are responsible for presenting a high quality caring [**13**]. Therefore, the manager’s ability development by the instruction of management skill could cause the increase of staff’s participating and their responsibility and the optimization use of personal’s capacity in the field of organization’s goals. For the aim of this study is to determine the impact of administrating skills instruction in the obstetrician’s efficiency in non-teaching hospitals which affiliated to the Kurdistan’s medical science university in 2015. 

## Methods

The present study is a quasi-experimental, which was done with the aim of determining the effect of management skills instruction to nurses and obstetricians managers on the obstetrician’s efficiency in the hospitals, which were affiliated to the Kurdistan’s medical science university in 2015. To avoid interaction and the effect of intervention group on the control group and getting better results, before sampling the hospitals separately classified to 2 control team and response one. Therefore, the researcher randomly divided them into two clusters in a way that three hospitals consider as a control group and three hospitals as an intervention group. The research’s sample constituted by all the nurses and obstetrician’s manager (matron, supervisors of the hospital and the matron who works in the department of obstetrics and gynecology) and the obstetrics and gynecology’s personals in hospitals which affiliated to the Kurdistan’s medical science university who have the criteria to enter the research. The obstetrics own, tenure, contractual and planning individual who has tended to participate in the study, working in the hospital at least six months before the research. Have associated degree or higher. Managers: tenure or contractual individual, having tend to cooperate, having a bachelor degree in nursing or obstetric and higher, having employment experience in the present post more than six months, having absentees in program management skill in the past one year. For determining the sample and proper and uniform distribution in hospitals, the classification sampling was used by knowing the number of obstetricians personal in each hospital and the number of samples was calculated based on the number of obstetricians in each hospital by using n = n N1/ N formula. In each group, 40 qualified obstetricians have entered the study. In this study, the sampling method for managers was a census. The efficiency standard questionnaire designed by Hersey, Blanchard, and Gold Smith in 1994 and its various reliability and validity have confirmed too. Heydar Abadi (2012) reported that the reliability coefficient of this questionnaire based on the Cronbach’s alpha for ability component was 0.805, for clarity was 0.818, for help and organizing support was 0.710. For evaluation was 0.855, for validity was 0.804, for incentive was 0.748, for the environment was 0.701, and the overall reliability coefficient of the questionnaire was 0.765. The surveys Cronbach’s alpha factor calculated 0.89 by Hatami et al. in 2011 and also the Cronbach coefficient was reported 0.92 by Davoodi et al. in 2008. Studying of the data collection has done by the self-reporting method. The data selection tool in this research was a 2-section survey. The initial section of the survey included demographic information (age, education level, work experience, the part which work in it, employment status, shift). The second part of it is related to the education of efficiency’s seven dimensions based on the ACHIVIE model (Hersi and Gold Smith), which contains the ability (3 question), recognition the job (4 question), help and support (4 question), incentive (4 question), performance feedback (4 question), manager’s decision (4 issue), environmental factor (3 question ). Each choice regulated based on the 5 Likert range the least [**1**], less [**2**], average [**3**], high [**4**], the highest [**5**]. 

Result’s interpretation: The maximum score for this questionnaire is 130. The nearest the total of the score to this number, the efficiency of the individual would be higher. Regarding the scores and the range of the Likert evaluation, the total of the scores could be judged and divided into 5 areas. It means that if the scores were 26 to 46, the efficiency was the least. If the total were 47 to 67, the efficiency was less. If the total were 68-88 the efficiency was average. If the total were 89-109 the efficiency was high and greater than this energy level was the highest. At the first phase, all the participant answers and the awareness consent was taken from them. Then the workshops were held at a particular time for managers who have the criteria to enter the study. Overall, from 25 qualified managers, a total of 16 managers (6 person obstetrician, and matron of gynecology ward and eight people supervisor and one person matron) participated in this management skill's instruction workshop. The samples separately have the management skill’s direction for 16 hours. is include an introduction, management principle, the definition of the managers and their task, solving problem’s power and group work, the communication skill and the main component of the communication skill and teamwork, leadership style time management, conflict management and incentive. Finally 12 weeks later finishing the intervention instruction, the survey again filled out by the samples (obstetrician’s personal in both case and control group). In the previous phase of the response 80 people in both groups completed the questionnaire and in the second step, 77 people answer the survey. The obtained results from efficiency’s questionnaire which filled out by obstetricians personal before and after holding the workshop for managers determined and compared. The results were explained by statistical SPSS software version 22 and variance analysis, independent and paired T-test. 

## Results

The study findings showed that more than half of the obstetricians were in the age group of 25 to 30 years old, and more than half of them have a work experience less than five years. For educational level, more than 90% of the obstetricians in both groups were expert and for their employment has of the obstetricians were employed, and half of them were passing their project period. For shifts, most of them have a circular shift, and half of the obstetricians in both groups were in gynecology ward and the other half were in a maternity ward. Two groups of the intervention and control were the same for average age, work experience, education level, and the department which work in it, employment status and shifts (**Table 1**). Based on the achieve model, the obstetrician’s efficiency scores were calculated. The efficiency’s average in both inversion and check group evaluated earlier and later inversion for each ability field, recognition the job, organization’s support, incentive, feedback, decision’s validity, and environmental factor. The obtained results from paired T-test indicated that there is a statistically notable distinct among the average rate of total efficiency in the intervention group in comparison with before intervention (P < 0.001). (**Table 2**). In the subgroup of ability, clarity, feedback, incentive, after the inversion, there is a clear variance among the rate of the knowledge field in both teams (P < 0.001). In the area of organization’s support, the validity of manger’s decision and environmental compatibility, despite the significant increase of efficiency score after the test, did not change, and remained average.

**Table 1:** The demographic variables and comparison of them between the 2 groups

Demographic variables		Intervention	Control	The results of statistical test
Age		28.92 ± 4.23	29.30 ± 4.46	T = -0.38, df = 78, P = 0.720
Experience	Less than 5 years	23 (57.5%)	25 (62.5%)	Chi = 0.26, df = 2, P = 0.87
	Between 5-10 years	13 (23.5%)	12 (30%)	
	More than 10 years	4 (10%)	3 (7.5%)	
The section which work in it	Maternity	21 (52.5%)	19 (47.5%)	Chi = 0.5, df = 1, P = 0.82
	Gynecology	22 (55%)	18 (45%)	
Shifts	Fixed	2 (5%)	3 (7.5%)	Fisher exact test = 1,000
	Circulating	38 (95%)	37 (92.5%)	
Employment status	Project period	22 (55%)	19 (47.5%)	Chi = 0.45, df = 1, p = 0.50
	employed	18 (45%)	21 (52.5%)	
Education level	Associate's degree	2 (5%)	1 (2.5%)	Fisher’s test = 0.708
	Bachelor's degree	36 (90%)	38 (95%)	
	Master's degree	2 (5%)	1 (2.5%)	

The results of the above table indicated that there was not a clear distinct for demographic variables among the two interventions and check team (P > 0.05).

**Table 2:** The average and nominal deviation and comparison of them before and after intervention

Group	After	Before		After		Paired T-test
		Average	SD	Average	SD	
Intervention	Ability	8.35	1.85	9.82	1.35	T = -8.51, df = 38, p = 0.00
	job’s recognition	9.82	1.90	12.82	1.50	T = -13.24, df = 38, P = 0.00
	Support	8.84	2.27	11.89	1.55	T = -11.10, df = 38, P = 0.00
	Incentive	7.10	2.37	10.28	1.80	T = -11.47, df = 38, P = 0.00
	Feedback	9.48	2.74	12.89	1.48	T = -7.48, df = 38, P = 0.00
	Validity	8.92	2.10	10.87	1.79	T = -7.50, df = 38, P = 0.00
	Environmental compatibility	7.82	2.25	8.84	1.61	P = -4.14, df = 38, P = 0.00
	Total score	60.35	8.35	77.43	5.90	P = -16.68, df = 38, P = 0.00
Control	Ability	8.23	1.65	8.05	1.75	T = 1.64, df = 38, p = 0.10
	job’s recognition	9.84	1.85	9.89	2.35	T = -0.13, df = 38, p = 0.89
	Support	9.39	1.65	9.34	1.64	T = 0.29, df = 38, p = 0.76
	Incentive	7.63	1.76	7.86	1.77	T = -1.38, df = 38, p = 0.17
	Feedback	9.55	1.76	9.55	1.70	T = 0.00, df = 38, p = 1.00
	Validity	8.86	1.61	8.84	1.66	T = 0.11, df = 38, p = 0.90
	Environmental compatibility	7.57	1.82	7.57	1.70	T = 0.00, df = 38, p = 1.00
	Total score	61.10	5.84	61.13	5.39	T = 0.04, df = 38, p = 0.96
Independent T-test	T = -0.24, df = 78, p = 0.80			T = 12.63, df = 75, p = 0.00		-

**Fig. 1:**
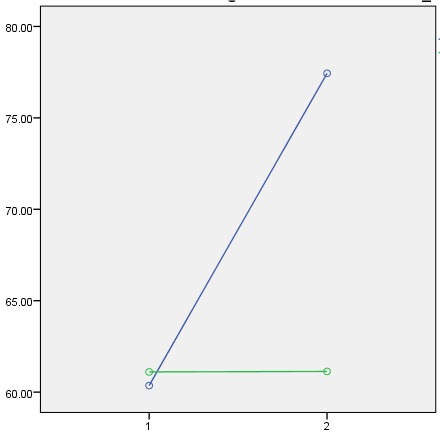
The comparison of efficiency score in the two groups of intervention and control

The results of **Table 2** showed that in the intervention team there was a clear distinct among total score and efficiency’s dimension earlier and later intervention (P < 0.05). In the control team, there was not a clear difference among earlier and later intervention (P > 0.05). Before intervention there was a clear variation among two group for their efficiency’s score (P > 0.05). After the intervention, there has been a clear distinction between the 2 groups (P < 0.05). The results of this study showed that holding the instruction program of management skill for nurses and obstetricians managers in hospitals caused the significant increase of the efficiency in obstetrician. The obstetrician’s efficiency levels before intervention were at a low level. The intervention group (blue line) has a significant increase in comparison with the control group (green line) (**Fig. 1**).

## Discussion and Discussions

Similarly, the Habashi Zadeh et al. study in 2011 which evaluated the effect of the professional competence promotion of nursing managers based on the nurse’s performance and efficiency showed that the average of the total energy in nurses before the intervention were at an average level but Sydin et al. [**14**]. Reported the effectiveness of the hospital’s personnel in Iran’s medical science university in a proper status which is not consistent with the present study. Qhamari Zadeh [**9**] study, which was done to determine the effect of sanitation management program on the leadership style, indicated that there is a clear distinction between the matron’s average effective score from the nurse’s point of view before and after the intervention. Hayli et al. believed that the managers should have the essential skills for having a successful organizational strategy, engagement with staff, and live answering to them [**8**]. In jobs which employees means more than income source, the efficiency promotion needs motivation in individual, and it showed the importance role of verbal and non-verbal communication among colleague, matron and service’s recipients. On the other hand, the researchers believed that even the active and fair feedback and encourages of managers could be a motivation factor in staff’s efficiency in a way that the team thought that their behavior observed, and the manager would see their loyalty [**15**]. Among the obstetrician’s demographic properties and their efficiency, there was not any relation in this study. This study was consistent with the results of Habashi Zadeh study (2012), but Letvak & Back in their study (2008) showed that there was a significant relation between the nurse’s properties such as age, work experiences, work shifts and their efficiency. In Fako study, there was a typical relationship between energy and age. The most qualified individual was in the age group of 35-44 years old and then 30-34 years old, then More than 45 years old and the least efficiency was in 25-29 and 20-24 old. The reason for these results is that the age group of 35-44 inanition to having experience, have energy too for doing their work in the best quality. The older person may become tired sooner, and the younger do not have enough experience [**16**]. Because problems which origin from low efficiency in health personal was attracted a lot of attention and may cause issues such as absentees in work and reduction of patient’s consent, the results of this research could help the nurses and obstetricians managers to prepare some way to have a proper work environment. For obstetricians, appropriate use of equipment and increasing the incentive of the obstetricians, which are the active factor on efficiency is necessary; the researchers believed that the educational level and job grade have not the primary role in the ability and the extra instruction period which related to the job is also necessary [**17**]. The unique introduction program which linked with long-term management could increase the management capacity which itself is a trump card for the competition with the organization [**18**]. According to the fact that the effect of the Directorate skill’s instruction program of nurses and obstetricians managers on efficiency evaluated, it has been suggested that some studies were well done in assessing the impact of other factors. Such as obstetrician’s sanitation, empowerment them, incentive factors, leadership style, etc. on the obstetrician’s efficiency. Also, a qualitative research should be done with this regard.

**Acknowledgements**

The researcher sincerely appreciates the obstetrics' personal and the hospital managers of the Kurdistan’s medical science university and the research assistant of that university. The researchers would also like to thank Tehran’s medical science university for financing the study and the distinguished professor of that university. 
